# Production of polyhydroxyalkanoate biopolymer from vinasse using *Ralstonia eutropha*

**DOI:** 10.1186/1753-6561-8-S4-P132

**Published:** 2014-10-01

**Authors:** Eduardo dos Santos, Claudia Gai, Kellen Zanfonato, Francielli Martinhago, Willian Steffen, Luci Kelin Quines, Melodi Schmidt, Willibaldo Schmidell, Glaucia Aragão

**Affiliations:** 1 Federal University of Santa Catarina, Department of Chemical Engineering and Food Engineering. Technological Center. Trindade, Florianópolis - SC - CEP 88040-900, Brazil

## Background

Brazil is the world's leading ethanol producer and exporter. Due to increasing fuel demands, ethanol production has escalated in recent years. Vinasse is generated as a byproduct of the ethanol industry and is mainly used as fertilizer. The excessive usage of vinasse in soil leads to groundwater contamination [1].

The objective of this project is to develop a fermentation process with the bacterium *Ralstonia eutropha *for the production of polyhydroxyalkanoates (PHAs) using vinasse as the main nutrient and carbon source. This process will enable the utilization of vinasse for production of bio-based, biodegradable P(3HB) biopolymer. These biopolymers are intracellularly accumulated by *R. eutropha *as carbon and energy reserves. The potential applications of P(3HB) as alternatives to petroleum-based plastics are abundant. In this work, pure vinasse was successfully used as substrate for the production of P(3HB) with the achieved accumulation reaching 50% of cell mass as polymer.

## Methods

The microorganism used was *Ralstonia eutropha *(DSM545).

Vinasse was collected at Usina Iracema (Iracemapolis, São Paulo, Brazil). A 50L sample was taken directly after the distillation process, sterilized by filtration and stored at -20ºC.

In order to analyze the P(3HB) production, two pre-cultures were prepared, one with NB and a second with vinasse with addition of urea [2].

Batch fermentation was done using a New Brunswick Scientific™reactor with a working volume of 4.0L, with no nitrogen feed. pH was corrected using 2.5 M NaOH and 2.7 M HCl solutions. Culture was followed during 22 hours.

Determination of biomass, nitrogen, and protein content were done according to [2]. The composition of the vinasse was determined by HPLC, as well as the final concentration of P(3HB), according to [3] with modifications.

## Results and conclusions

Previous results from our group suggested no inhibition effect of vinasse on *R. eutropha *growth and the strain can grow with a maximum growth rate of 0,27h^-1.^

The analysis of the vinasse revealed that its main components are glycerol, fructose, saccharose and mannitol. These substrates can be utilized by the bacteria. According to the experimental analysis, the microorganism barely used fructose to grow and accumulate biopolymer. Saccharose and mannitol, on the other hand, were consumed entirely. 25% of the glycerol was consumed.

The nitrogen concentration when the culture started was 0.063 g.L^-1^, which indicates nitrogen limitation since the beginning of the culture. As a consequence, P(3HB) was accumulated during all culture to a concentration of around 50% during the culture. This is low compared to 80% (gPHB/gBiomass) in defined media [4] but promising from a waste product. According to the carbon balance, 79% of the calculated biomass and biopolymer production was reached from vinasse, indicating the feasibility of the process.
